# Emergence of integrated institutions in a large population of self-governing communities

**DOI:** 10.1371/journal.pone.0216335

**Published:** 2019-07-11

**Authors:** Seth Frey, Robert W. Sumner

**Affiliations:** 1 Communication Department, University of California Davis, Davis, CA, United States of America; 2 Neukom Institute for Computational Science, Dartmouth College, Hanover, NH, United States of America; 3 Computer Science, ETH Zurich, Zürich, Switzerland; City University London, UNITED KINGDOM

## Abstract

Most aspects of our lives are governed by large, highly developed institutions that integrate several governance tasks under one authority structure. But theorists differ as to the mechanisms that drive the development of such concentrated governance systems from rudimentary beginnings. Is the emergence of integrated governance schemes a symptom of consolidation of authority by small status groups? Or does integration occur because a complex institution has more potential responses to a complex environment? Here we examine the emergence of complex governance regimes in 5,000 sovereign, resource-constrained, self-governing online communities, ranging in scale from one to thousands of users. Each community begins with no community members and no governance infrastructure. As communities grow, they are subject to selection pressures that keep better managed servers better populated. We identify predictors of community success and test the hypothesis that governance complexity can enhance community fitness. We find that what predicts success depends on size: changes in complexity predict increased success with larger population servers. Specifically, governance rules in a large successful community are more numerous and broader in scope. They also tend to rely more on rules that concentrate power in administrators, and on rules that manage bad behavior and limited server resources. Overall, this work is consistent with theories that formal integrated governance systems emerge to organize collective responses to interdependent resource management problems, especially as factors such as population size exacerbate those problems.

## Introduction

The Internet has empowered normal people to easily craft and deploy whole social systems, and to attract and maintain vibrant communities of total strangers. As online communities thrust millions around the world into the shared management of such artificial "resource systems", these unwitting amateur governors find themselves struggling to manage a disparate bundle of social dilemmas. Wikipedia, for example, must reduce vandalism from malicious edits, retain quality editors, and solve the public goods problem of soliciting contributions to a service that is free to all. Studying communities' failures and successes promises not only to advance fundamental questions about resource governance [1,2], but to cultivate in citizens a native comfort with the skills, in both governing and being governed, that demanding institutional forms like democracy require [3].

Understanding success in online communities is in large part understanding the development of stable formal governance systems from rudimentary beginnings. What drives institutions to expand from individual collective action problems and consolidated themselves into bodies that integrate operations over complexes of governance tasks? Some political and organizational theories characterize the expansion of institutions beyond simple idealized forms as a symptom of organizational pathologies, as special interests capture governance mechanisms, or historical accidents accumulate inefficiently [4–6]. These theories, typical of anarchist and libertarian thought, see formal governance as emerging to institutionalize the power of small status groups, or more efficiently extract rents. A less cynical view, the theory of "institutional diversity," holds that governance institutions grow in size and complexity in order to expand their purview and more effectively respond to interdependent environmental demands [7,8]. This latter idea, an application of cyberneticist W. Ross Ashby's Law of Requisite Variety [9,10], captures an observation from decades of fieldwork in forests, fisheries, pastures, and irrigation systems, that the most successful and enduring self-governing resource management institutions are those that use a variety of rule types, that integrate a range of collective tasks under one institutional structure, and that distribute authority across many levels of that structure [7,11]. The thesis of this perspective is that the resource management systems that communities develop are as complex as they need to be to adapt to their socio-ecological circumstances [12].

Insights into the evolution of governance complexity are necessary for understanding effective policies and organizations. And questions around the origin and success of complex, integrated, or formal institutions are of immense theoretical interest across the social sciences, in the institutional, historical, and organizational subdisciplines of economics [13–15], anthropology [16], political theory [17,18], organization theory [19], from animal behavior [20] to complex systems [21–25]. In pursuit of these theories, empirical researchers have benefitted from simple artificial norm systems that focus on a single problem [26,27] and, at the other extreme, from individual quantitative case studies of mature multifaceted institutions [28–30]. But closing the explanatory gap between these extremes—explaining the development of fully developed institutions from rudimentary emergent norm systems—is a forbidding challenge, one that demands population-level comparisons across many independent but comparable institutions [1,31–35]. Independent hosts of Wikipedia's MediaWiki software platform have been an especially fruitful domain for comparative system-scale analysis, with large scale comparisons illuminating questions around structural predictors of successful online communities, the emergence of oligarchy in online peer production systems, and their life histories [36–38]. Virtual social systems like peer production platforms, forum communities, and multiplayer game worlds are appealing because they are relatively simple, highly replicable, easily scrapeable, and they attract motivated people pursuing clear goals under knowable constraints. Our analysis of the rule systems of 5,216 virtual communities within one popular online platform contributes to an understanding of comparative institutional design by revealing correlates of community building success, and suggesting mechanisms for those relationships in terms of the theory of institutional diversity. Our approach, an automated population-level comparative analysis of standardized sociotechnical systems, is naturalistic and yet constrained enough by theory and in form to be amenable to causal interpretation.

## The resource management perspective

The frameworks of natural resource management are valuable for understanding successful administration of online communities. We focus on the frameworks of the Ostrom Workshop, which have been used to study other kinds of online communities [39–43].

Community building, online and in general, is attended by a number of collective action problems that derive from the special properties of different resources, particularly the properties of non-excludability and subtractability. A non-excludable resource is one that is not subject to private ownership in the sense that an agent cannot prevent others from accessing or consuming that resource. A subtractable resource is one that is finite: consumption by one agent constrains the potential consumption of it by another. Common pool resources are defined as non-excludable and subtractable [8], and examples include common pastures, shared office kitchen sinks, and global fish populations. They are vulnerable to over-extraction via the mechanism of the Tragedy of the Commons [44], a property that unifies otherwise disparate commons such as fisheries, forests, and irrigation systems [32]. Public goods are also non-excludable but they differ from common pool resources in being defined by their non-subtractability. Examples include breathable air in a room, radio broadcasts, uncongested public roads, and not-for-profit online information sources. Because they are not limited, the social dilemma commonly associated with public goods is less concerned with regulating access than with ensuring sufficient provisioning. Public goods often require maintenance, but the same incentives that drive individuals to over-extract common pool resources drive them to neglect public goods [45]. Public bads are a type of resource with the same properties as public goods except that provisioning tends to be costless and their externalities are negative rather than positive; public bads are pollutants, and the collective action problem associated with them is pollution, costless to produce, costly to manage, and affecting everyone. An effective institution will solve public bad provisioning by minimizing its costs or discouraging the behaviors that produce it.

Understanding a community's resources in these abstract terms makes community success amenable to frameworks for analyzing real world community resource management institutions such as local fisheries, irrigation systems, and forest management communities. These systems differ dramatically in their social and ecological particulars, but researchers have succeeded at articulating theories and approaches that make lessons from one generalizable to others. It is from these generalization efforts that we have a basis to argue for the generality of our conclusions: as online server communities can be treated as resource management institutions that manage familiar resource types, and mitigate familiar problems, the factors that lead to their success are promising candidates as predictors of success in other resource settings.

## The challenge of governing a sovereign online community

The millions of sovereign self-governing communities on the Web provide an unprecedented opportunity for comparative insights into institutional development over multiple scales of population [35,46,47]. Any person with an Internet connection can follow simple templates to build a global community around common interests. And just as in a physical community, success online depends on a community's ability to overcome collective action problems of longstanding scientific interest [11,44,48]. In this sense, online communities are self-organized resource governance systems that are replicable, and that can advance existing theories of institutional success [32].

Over a period of two years, we monitored over 5,000 web servers hosting instantiations of a popular online community platform (described in detail in the Methods and S1 Text), in order to compare the success of their communities. Server communities in this system are all sovereign and independent from each other, yet they face common resource management problems that put them under intense selection for survival. They face daunting resource constraints that are amplified by population growth. They share standardized interfaces with the outside world, yet are customizable enough to permit vast individual differences in culture and management style. And they are all led by their founding server administrator, who by default makes all governance choices unilaterally and bears all time and money costs. In this setting, success means recruiting, supporting, and retaining a core group of devoted community members and fostering successful collective action among them. In order to attain it, an administrator must overcome the resource challenges that cause communities to fail.

After an administrator specifies the desired size of their community (ranging in our data from the single digits to the hundreds), they are responsible for managing three types of public resource: "virtual" resources defined by the software platform (such as software-based currency or reputation systems), physically constrained resources (such as limited RAM, CPU, bandwidth, and monthly server fees), and the consequences of antisocial behavior (such as vandalism, harassment, and hacking). These types span resource categories to include common pool resources, public goods, and pollution, all of which pose problems for collective action, the first two because they must be maximized, and the latter because it must be minimized [11]. These resources are very difficult to manage well. Vandalism and other forms of pollution are endemic to the platform, increasingly so in larger communities. And because the software is so resource intensive, a basic server can become strained with as few as 2 users, much less 200. Recommendations to server owners suggest that a server be provisioned with several Mbits of network bandwidth and 1 GB of RAM *per additional player* in order to provide users with a sufficiently responsive experience. In the face of CPU, RAM, and bandwidth limits, the only alternative to constraining user freedom is assuming the costs of operating a more powerful machine, and either providing it for free or looking to users for revenue streams such as donation drives, membership fees, paid advertisements, or premium services. On top of this, each community exists in a larger ecosystem of communities that are in competition for a fourth scarce resource, committed users, who are in demand because of the great potential value they can bring to the communities that manage to retain them. For example, in a remarkable investigation of competitive practices among professional video game servers, security researchers described hacker-for-hire schemes in which competing server administrators contract denial-of-service attacks designed specifically to frustrate and fragment their competition's user base [49]. These exploits are uncommon in the amateur-run (non-professional, not-for-profit, and generally smaller) servers we focus on in this work. Nevertheless, they successfully illustrate the sensitivity of server communities to resource challenges: an administrator who fails to provide necessary resources up to users' standards, whether physical or virtual, risks the desertion of their core group to a better-run server.

Fortunately, online communities are in a unique position to benefit from self-organized governance systems—formalized text policies and automated software rules. A study of Wikipedia's formal policy shows strong conservation of core rules, around which subsequent rule-making has organized itself [25]. In the communities we study, the challenges posed by resource-related social dilemmas have been made approachable with open-source catalogs of "plugins": modular programs that automatically implement rules and other political-economic constructs. These include temporary bans, full exile and blacklisting, hacking counter-measures, cheater detection, peer monitoring and reporting tools, surveillance tools, distribution of authority to trusted members, tools that reduce the number of game actions to those that are least computationally costly, and tools for keeping and restoring backups after failures and attacks. A plugin called Lockette instantiates private property to prevent stealing by implementing the idea of personal storage, one called WorldBorder conserves server disk resources by making virtual resources more limited, and another called LogBlock improves monitoring of all three types of resource by giving every possible virtual entity a public recorded history. Code in these communities is literally law [50]. And by "mixing and matching" many such rule systems, an administrator can implement virtually any governance system. In this work, we represent an administrator's governance style as a distribution over the types of governance plugin they have installed.

## Measures and predictions

In the communities we study, administrators start from a default state of a near absence of rules, and approach several types of resource challenge by selecting across several types of rule system: those that facilitate interpersonal communication, information diffusion, resource exchange, and top-down administrator control. All of these types have been implicated in improving outcomes in economic and collective action institutions [51–54].

Another key choice that administrators make in this platform is deciding the value of a mandatory server parameter: the maximum number of users who can be logged in simultaneously. We use this setting as a proxy for each administrator's desired target population size.

**Fig 1:**
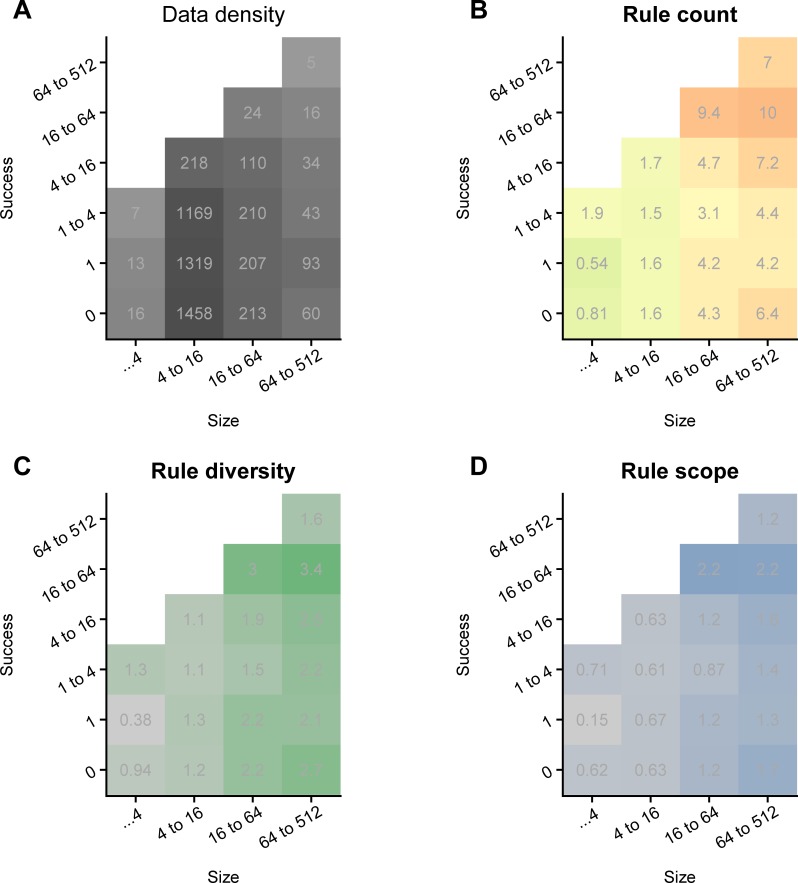
Most communities are small and unsuccessful. Larger successful communities have more rules governing more kinds of resources. We analyze 5,200 amateur-run web server communities. Each server is operated by an administrator who makes all governance decisions. Among these decisions is the server *size* (*x-axis)*, the server's maximum number of users who may participate at any moment. This number represents an administrator's desired community size and puts a practical upper bound on the community's core group or *success*: the number of users who return to the community regularly (*y-axis; all plots*). Beyond return visits, unique monthly visits to many of these communities exceed the thousands. **A.** We summarize the data in a 2D histogram of all communities binned by success and size, with each bin reporting the number of communities within the given range, and marginals represented by grey ticks. Most communities have size 4–16, and most fail to grow a core group larger than one. The most interesting communities, those with the largest core group for their class, are along the diagonal upper edge of each plot. A bin's shade of grey, its number label, and the marginals all communicate the same distributional information redundantly: the count of communities by size and success. **B.** Administrators select their community's governance regime by installing combinations of software modules that implement rule systems. This panel shows the mean number of rules in use by communities in a bin. **C.** and **D.** All rules address some resource problem with some kind of rule. There are different problems and different rules (Fig 2), and we plot diversity metrics over them. Panel C shows that large successful communities use a greater variety of rules types ("rule diversity"). Panel D shows that they attend to a greater variety of resource problems ("rule scope").

We define community success as the size of a server's "core group," the number of users who returned at least once a week for at least four consecutive weeks. In contrast to raw number of visits over a time period, return visits indicate a sustained level of interest and commitment (S1 Text). By measuring core group size with target size in mind, we define "success" with respect to administrator goals (Fig 1): a community with a core group of 4 (4 users who returned at least once a week for a month) is successful if its target size is 4, and not if its target size is 100.

**Fig 2:**
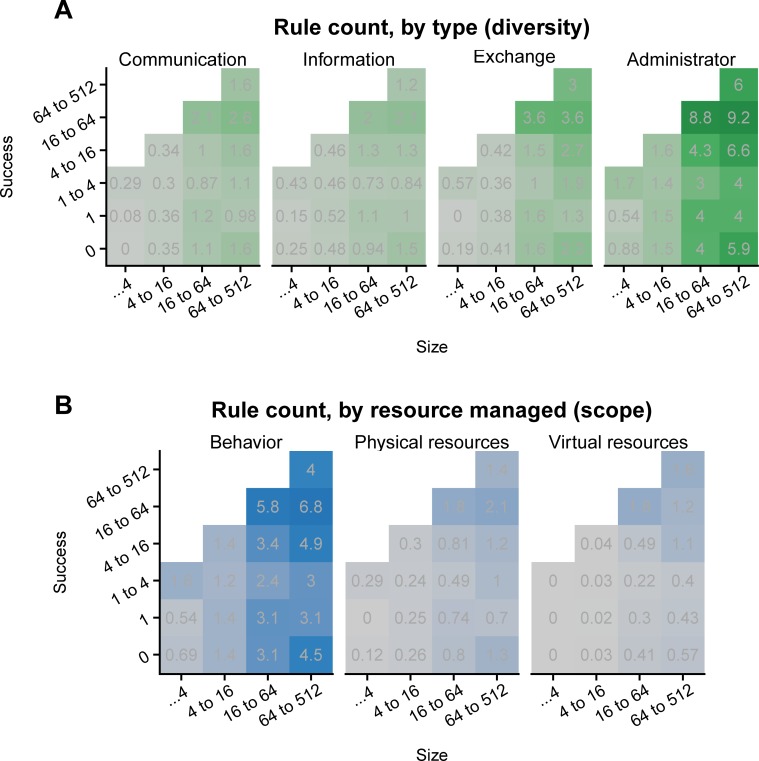
Larger successful communities use rule systems with more types of rules governing more types of resources. Actively managing physical server resources increases success with size. Each plot shows the mean number of rules per bin, per rule type (**A**) or target resource (**B**). The most common type of rule extends an administrator's power over their server. The resource challenge that attracts the greatest number of rules is the management of bad behavior. These two types, as well as rules that manage physical resources, increase in use significantly with population maximum (p<0.001). As their shared colors indicate, Fig 1C shows the diversity across the 4 plots of A, and Fig 1D represents the data over the 3 plots of B. For reference, both rows of figures roughly sum to Fig 1B.

The 5,216 independent communities in our dataset represent a wide range of sizes, with 1–30,000 confirmed visits per month, 1–3,100 unique visits, target size from 2–284 (median 6), and success (in terms of core group size) ranging from 0–400 (median 1). The median lifetime of these communities was 8 weeks.

With multiple communities of multiple orders of magnitude all overcoming similar problems in a competitive setting, this platform lets us test the relationship between institutional structure on governance success, as moderated by target population size. Following the claims of the theory of institutional diversity, that institutional complexity is a response to environment complexity [10,55], we measure how communities develop in terms of three dimensions of regime complexity: the number of rule systems they install (rule count), the heterogeneity of rule types they represent (rule diversity), and the purview of those rules, in terms of number of resource problem types (rule scope). Rule count gives a fairly literal representation of the size or extent of a server's policy apparatus, complexity in the sense of representing a large complex of rules. Rule diversity is the closest indication of institutional diversity: the variety of structures and rule types that an institution employs. For example, ideologically market-focused or authority-focused administrators might try to build servers that rely only on plugins that institute exchange mechanisms or that further empower the administrator. Their servers would show low institutional diversity, while a less discriminating or more pragmatic server with a variety of rule types would show high institutional diversity. Rule scope indicates the number of resource challenges that the administrator is explicitly attending to; are they focusing narrowly on just the problem of bad behavior, or server resources, or are they working in parallel to manage many? All three complexity measures capture facets of an institution's development or integration, with rule scope and diversity respectively capturing the complexity of the types of problem and solution, and rule count capturing the extent of the institution. As a fourth measure, we also calculate rule specialization, which indicates how unique a community's governance was in the population of communities, as a proxy for the role of niche size, a concept from organizational ecology [56].

Varying in size from a few members to thousands, in the governance choices that their administrators have made, and in the levels of success they have achieved, this population of amateur-run servers makes it possible to observe formal institutions in various stages of development. What governance features predict a community's success, and how do those features differ between large and small communities? Do larger or more successful communities have more developed or complex governance schemes? If the drivers of institutional development are historical accident and capture—an accumulation of vestiges and institutionalization of arbitrary power—than we shouldn't expect more complex governance regimes to be more successful, but we might expect complexity to increase with size. We would also expect complexity to increase in size under the competing theory, that complex, integrated regimes develop in response to the demands of a complex, interdependent environment. But even though an effect of size does not distinguish the theories, they do differ in their predictors of success. Under the institutional theories of the resource management perspective, institutions will be successful to the extent that they can respond effectively to the demands of their environment, and the environment will become more demanding as resource limits impose more onerous constraints on population size. Following both theories, we predict that all measures of complexity (rule count, rule diversity, and rule scope) will increase with increases in community population. Following the resource management literature, we also predict that these measures will increase with success, particularly among larger communities.

## Data and methods

The online communities in our population are all servers of the multi-player "virtual world" video game Minecraft. Previous research with the game has focused on individual or group level game behavior, with a focus on creative play, collaboration, and engineering applications [57–61]. For our purposes, Minecraft stands out less for its qualities as a game *per se*, and more for the ecosystem of servers, tools, players, and practices that the player community has collectively built around it. By contrast to the business models supporting other games, where all servers are managed by a single professional entity, playing Minecraft with others usually means logging into an openly accessible server, somewhere in the world, that is being provided by an unpaid amateur without professional experience in governing strangers or managing server resources. Minecraft is an ideal domain for comparative institution-scale analysis because it is one of few games with a decentralized amateur-driven hosting model and a large user base. And it is ideal for testing questions of resource management and economic governance because administrators have autonomy, a clear goal, a wide variety of tools, and a challenging resource environment. Independent of the game's specifics, merely logging in imposes a substantial burden on that server's computational resources, one that threatens to undermine the game experience for all. If the difficult nature of the bounded resources were not enough, the population also poses challenges. Most players are anonymous and often immature youth, two qualities that should make governance more challenging for a server administrator [62,63], and correspondingly more interesting for the study of successful resource management institutions.

Our analysis was based on a dataset of API queries from 370,000 Minecraft servers contacted between 2014/11 and 2016/11, several times daily. By default, these servers are publicly accessible via the Internet and do not have terms of use. Our scraper accessed each community for several public server performance statistics, including rules installed, maximum simultaneous users allowed (server "size"), and the anonymous IDs of users present. After filtering out disconnected servers (~220,000), those that did not survive for at least one month (~70,000), and those that did not report full governance information (~75,000), we had a corpus of 5,216 minimally viable, minimally comparable online server communities, 1,837 of which were also minimally successful (full detail in S1 Text). Part of minimum comparability is that we excluded large professional servers from our analysis, chiefly because their ultimate goal is not to build a community but to be profitable. This difference leads them to work to maximize impressions (unique rather than return visitors) and to focus on distinguishing themselves from other large servers, modifying the game environment and mechanics so heavily that they are scarcely recognizable as servers of Minecraft, in terms of the challenges they face or how they address them.

Administrators select software rules from a single central community-managed plugin repository. Within this system, each is assigned by its author to a category that describes what type of rule it is. We used these categories to classify rules into types, and to count each community's rules by its governance characteristics. A community's rule count is the sum of plugins over all three resource types. A community's rule diversity and rule scope (resource diversity) are the ecological variety (number of types) represented by its total system of rules: a server with no governance plugins has ecological variety of zero, while a server with at least one plugin in two different categories has variety two. A server's rule specialization was the median, over all plugins, of the number of other servers that plugin was observed on.

Our main analyses regress core group size and the *log*_*2*_ of population maximum against these four measures of institutional diversity and the interactions of each with community target size, and several basic covariates (Tables 1 and 2, S1 Text). To cancel the leverage that unsuccessful communities had on models of population size (which did not control for core group), we conducted all tests on population maximum on only the subset of 1800 minimally successful communities (core group size > 1).

**Table 1:** Models of population size. Rule count and other measures of regime complexity are greater in larger minimally successful communities.

	Dependent variable					
	Pop. max.					
	*(1) controls*	*(2) ctrls+1feat*	*(3) ctrls+1feat*	*(4) ctrls+1feat*	*(5) ctrls+1feat*	*(6)* *full*
Intercept	3.65*** (0.25)	3.65*** (0.24)	3.58*** (0.25)	3.65*** (0.25)	3.64*** (0.25)	3.73*** (0.24)
API richness	-0.31*** (0.07)	-0.31*** (0.07)	-0.31*** (0.07)	-0.31*** (0.07)	-0.33*** (0.07)	-0.32*** (0.07)
Software count	0.02*** (0.001)	0.01*** (0.001)	0.02*** (0.001)	0.02*** (0.001)	0.01*** (0.001)	0.01*** (0.001)
Week	1.49*** (0.15)	1.24*** (0.15)	1.35*** (0.15)	1.49*** (0.15)	1.29*** (0.15)	1.22*** (0.15)
Weeks up	-0.02*** (0.002)	-0.02*** (0.002)	-0.02*** (0.002)	-0.02*** (0.002)	-0.02*** (0.002)	-0.02*** (0.002)
Rule count		0.09*** **(0.01)**				0.11*** **(0.02)**
Rule diversity			0.13*** **(0.02)**			–0.14*** **(0.04)**
Rule specialization				0.63 (0.80)		1.00 (0.78)
Rule scope					0.28*** **(0.03)**	0.15* (0.07)
Observations	1,837	1,837	1,837	1,837	1,837	1,837
R^2^	0.19	0.24	0.21	0.19	0.22	0.24
Log Likelihood	–2,953	–2,897	–2,929	–2,952	–2,916	-2,890

*p<0.05

**p<0.01

***p<0.001.

A server's max. population size is the maximum number of users who can be logged in simultaneously, and it proxies the administrator's desired community size. Columns report regressions fitting features of 1,837 minimally successful communities to log_2_ of population size. Predictors of interest are high-level features of the rule systems installed by communities. Model 1 fits only controls, models 2–5 fit each institutional feature individually, and model 6 fits all predictors. Control variables include the richness of a community's voluntary public API reporting, its total number of installed plugins (both governance related and non-governance related), a date of the server's measured activity in weeks, and its duration to date, in weeks.

**Table 2:** Predictors of community success, in terms of core group size. Effect of rule count and other measures of regime complexity interacts with population size.

	Dependent variable					
	Core group size					
	*(1) controls*	*(2) ctrls+1feat*	*(3) ctrls+1feat*	*(4) ctrls+1feat*	*(5) ctrls+1feat*	*(6)* *full*
Intercept	-0.97*** (0.15)	-0.73*** (0.15)	-0.90*** (0.16)	-0.97*** (0.15)	-0.85*** (0.16)	-0.84*** (0.15)
API richness	0.01 (0.04)	-0.02 (0.04)	0.005 (0.04)	0.01 (0.04)	-0.01 (0.04)	0.01 (0.04)
Software count	0.01*** (0.001)	0.01*** (0.001)	0.01*** (0.001)	0.01*** (0.001)	0.01*** (0.001)	0.01*** (0.001)
Week	0.17 (0.09)	0.18* (0.09)	0.19* (0.09)	0.17* (0.09)	0.18* (0.09)	0.17 (0.09)
Weeks up	0.01*** (0.001)	0.01*** (0.001)	0.01*** (0.001)	0.01*** (0.001)	0.01*** (0.001)	0.01*** (0.001)
Pop. max.	0.27*** (0.01)	0.25*** (0.01)	0.27*** (0.01)	0.27*** (0.01)	0.26*** (0.01)	0.26*** (0.01)
Rule count		-0.01* (0.01)				0.0002 (0.01)
Pop. max. × Rule count		0.02*** **(0.003)**				0.03*** **(0.005)**
Rule diversity			-0.03** (0.01)			-0.06* (0.03)
Pop. max. × Rule diversity			0.01* (0.01)			-0.05*** **(0.02)**
Rule specialization				-0.57 (0.47)		-0.48 (0.46)
Pop. max. × Specialization				-0.10 (0.16)		0.02 (0.16)
Rule scope					-0.04 (0.02)	0.05 (0.05)
Pop. max. × Rule scope					0.04*** **(0.01)**	-0.02 (0.03)
Observations	5,216	5,216	5,216	5,216	5,216	5,216
Log Likelihood	-8,494	-8,472	-8,489	-8,492	-8,488	-8,449
Deviance	5,190	5,200	5,189	5,190	5,191	5,208

*p<0.05

**p<0.01

***p<0.001.

A server's core group size is the number of users who returned at least once a week for a month, and it quantifies success at community building. Columns report negative binomial regressions fitting features of 5,216 communities to core group size. Predictors of interest are high-level features of the rule systems installed by communities. Model 1 fits only controls, models 2–5 fit each institutional feature individually, and with its interaction with population size, and model 6 fits all predictors.

## Results

Although our analysis is correlational, we present a causal reading of our results. Specifically, we interpret our results as estimating the effects of governance regime as a necessary condition for success. We support this interpretation on the grounds that domain constraints prevent a community from growing its core group without having overcome endemic resource problems. Plentiful resources are a prerequisite to the successful cultivation of a large core group. As large population sizes exacerbate resource management problems [48], communities are under more pressure to manage resources well. It is important to keep in mind the difficulty of collective action in this setting and others [32,64]. Within our own sample, only 35% of administrators ever recruit a core group larger than themselves, and fewer than 5% build a core group of more than four. With more users in-game resources are extracted at a more aggressive rate, server CPU, RAM, and bandwidth approach their physical limits, and the probability of malicious users increases. Any one of these may drive community members away. With the intense selection pressure on this population of communities, especially large ones, it is not feasible for servers to attain our definition of community building success without having already achieved success at managing all of the resource problems that constrain their growth.

We first consider how governance features are correlated with server population size. As this value is actually a desired maximum set by each administrator, predictors of server size tell us how administrators' understandings of effective governance style change with their intended community size, among communities that were at least minimally successful. As the governance features of interest are likely to covary, and we were unconstrained by theories for which features might affect which others, we ran several models, building up from models that test each feature individually (plus controls), to a full model including all features. Considered together, the single-variable models are consistent with the interpretation that the three complexity measures all correlate positively with maximum server size (tested separately; Fig 1; all *p*<0.001; Table 1 models 2–5) and that rule specialization does not (*p* = 0.43). The full model (Table 1 model 6) complicates this picture only slightly. According to both the single and full models, rule count has a very robust positive correlation with server size (Table 1 models 2 and 6), suggesting that minimally successful servers consistently install more governance plugins as their target size increases. The full model also supports the lack of effect of a server's specialization, in terms of the uniqueness of its rules relative to those in use by other servers (*p* = 2.0), and complicates the interpretations rule scope and rule diversity, the first of which is effectively insignificant considered in combination with the other features (*p* = 0.03), and the second of which remains significant, but flips from positive to negative, suggesting that rule diversity actually decreases with server size (*p*<0.001). These results are likely due to covariance patterns between diversity and scope, which in turn may give hints as to the mechanisms behind the correlations of these features with maximum server size.

Given an overall increase of institutional complexity with intended server size, we also consider the effects of these same features on community success, as operationalized by the size of a server community's core group (again, the number of visitors who returned to the community at least once a week over a month; Fig 1 and Table 2). In order to capture both the overall effect on success, and any contingencies of these effects with size, our models fit both main effects and interactions with maximum server size. Socioecological theories, such as those from the resource management perspective, predict a positive relationship between governance complexity and community success in such a complex resource setting, one that we interpret directionally as a positive effect of complexity on success.

We find that several indicators of institutional complexity are statistically significant predictors of community success, but only in interaction with a server's maximum population size. In the models testing governance features individually, the main effects of rule count, diversity, scope, and specialization on success are above our *p*<0.001 threshold for statistical significance (*p* = 0.02, *p* = 0.005, *p* = 0.06, *p* = 0.22; Table 2 models 2–5). However, considering interactions with community size reveals significant positive effects of two features in interaction with size: rule count and rule scope (both *p*<0.001; models 2 and 5). Deploying a greater overall amount of formal (code-mediated) rules increases success among larger servers, as does deploying rules that address a greater variety of resource types. Considering the full model (Table 2 model 6), with all four governance features together, again supports the overall robustness of the positive effect of rule count in interaction with size (*p*<0.001), the clear insignificance of rule specialization (*p* = 0.87), and a colinearity relation between rule diversity and scope that may result from the specific mechanisms connecting regime style to success. In the full model, rule scope becomes insignificant (*p* = 0.34) and the effect of rule diversity on success with size becomes significantly negative (*p*<0.001).

We next look more closely into rule scope and rule diversity, to determine if certain rule types, or attention to certain types of resources, are significantly associated with size or success (Fig 2). Within rule types, we find that larger communities rely to a greater extent on rules that further empower a server's central administrator (*p*<0.001, Table 3 model 1), but no significant effects on community success, either alone or in interaction with size (Table 3 model 2). Investigating within resource types we find statistically significant positive correlations between size and rules for behavior and physical resources (both *p*<0.001, Table 4 model 1). Physical resource management rules also have a significant positive effect on success, but only in interaction with size, such that larger servers that focus on the governance of computational resources are significantly more successful (*p*<0.001, Table 4 model 2).

**Table 3:** Rule types as predictors of population size and core group size. Rules that empower administrators are more likely among large servers.

	Dependent variable	
	Pop. max.	Core group size
	*(1) rule types*	*(2) rule types*
Intercept	2.91*** (0.04)	-0.59*** (0.04)
Software count	0.01*** (0.001)	0.01*** (0.001)
Pop. max.		0.25*** (0.01)
Rule_Communication_	0.09 (0.05)	-0.04 (0.03)
Rule_Information_	-0.09 (0.05)	0.03 (0.04)
Rule_Exchange_	0.01 (0.05)	0.01 (0.03)
Rule_Administrator_	0.11*** **(0.03)**	-0.02 (0.02)
Pop. max. × Rule_Communication_		0.0002 (0.02)
Pop. max. × Rule_Information_		0.01 (0.02)
Pop. max. × Rule_Exchange_		0.03 (0.02)
Pop. max. × Rule_Administrator_		0.001 (0.01)
Observations	1,837	5,216
R^2^	0.21	
Log Likelihood	-2,936	-8,540

*p<0.05

**p<0.01

***p<0.001.

Note that maximum population size is the dependent variable in the first model and an independent variable in the second.

**Table 4:** The resources targeted by rules, as predictors of population size and core group size. Rules that manage a server's computational resources are increasingly successful with size.

	Dependent variable	
	Pop. max.	Core group size
	*(15) resource types*	*(16) resource types*
Intercept	2.93*** (0.04)	-0.55*** (0.04)
Software count	0.01*** (0.001)	0.01*** (0.001)
Pop. max.		0.24*** (0.01)
Resource_Grief_	0.08*** **(0.01)**	-0.03** (0.01)
Resource_RealWorld_	0.42*** **(0.05)**	0.03 (0.06)
Resource_InGame_	0.03 (0.04)	0.01 (0.02)
Pop. max. × Resource_Grief_		0.004 (0.01)
Pop. max. × Resource_RealWorld_		0.08*** **(0.02)**
Pop. max. × Resource_InGame_		-0.01 (0.01)
Observations	1,837	5,216
R^2^	0.22	
Log Likelihood	-2,924	-8,521

*p<0.05

**p<0.01

***p<0.001.

Note that maximum population size is the dependent variable in the first model and an independent variable in the second.

Under our interpretation, a community's complexity or specific style of governance has increasing influence on its likelihood of succeeding or failing as it aspires to be larger. Because larger populations exacerbate each of the major resource problems facing servers, large communities are more susceptible to spontaneous failures of collective action, and seem to require more intentional, complex, integrated governance in order to succeed at recruiting and maintaining a sizeable core group. Overall, our results are consistent with resource management theories that complex governance schemes emerge in response to the demands of complex resource environments.

## Discussion

What drives small, rudimentary informal institutions to develop themselves into integrated, formal governance systems? In the context of online communities, the increases we find in the number and scope of rules with success are consistent with institutional diversity's prediction that regime complexity emerges among successful servers as an adaptation to a complex environment. And our context-sensitive definition of "success," as relative to each community's own target size, implicates population size as a major driver of regime complexity.

Our findings also support the idea that communities benefit from a strong administrator. Although the ideas of community and small-scale governance often imply democratic aims, the communities we study here are not democracies. By default, a single administrator maintains complete control and, as we show, their power and authority increases with size. While it may be tempting to interpret this as an emergence of autocratic rule, it is important to be mindful of the evolutionary dynamic that binds these communities: communities actively compete for users, and users have unrestricted freedom to "vote with their feet" at negligible or relatively low cost (S1 Text) [65]. While their investments in a community may increase their apprehension at leaving, or their tolerance for bad administrators, the stakes of abandoning game constructs is ultimately small relative to the stakes that people face in more familiar applications of market theories of governance: the choice of what city to move to, what nation to immigrate to, or what representative to elect. Furthermore, players' IDs persist across servers, making it possible for them to retain certain types of value across servers, such as friendships and social capital. The persistence of player IDs makes it much easier for players to maintain relationships across servers.

Under the competitive conditions that servers endure, the best explanation for the value of concentrated authority is that users prefer it, and select into servers that exhibit it. Because we, like others [7,66,67], are liable to romanticize the decentralization of political authority, this finding reminds us of the value of leadership in collective action settings, and it favors various economic theories of the state [15,68], including the utopian thesis of Nozick [4] that when tyrants must compete, market forces can drive them to govern as if they were benevolent. Therefore we propose that the relationship of increased administrator power to community success may not reflect an increase in, or preference for, authoritarianism.

### Limitations

The resource management framework we use, developed by the community of scholars around Elinor and Vincent Ostrom, were developed precisely so that insights from one socioecological setting, such as common pasture, could generalize to vastly different settings—fisheries, forests, irrigation systems, or online communities—despite the peculiarities of each [32,69]. In all of these domains, and in Minecraft as well, environmental conditions create social dilemmas around valuable resources, and agents with high motivation and clear goals create institutions whose proper functioning aligns private goals with the public good. In these general terms, a multiplayer video game like Minecraft is as valid a source of generalizable insights as any other ecosystem. Still, there are bound to be properties of virtual communities that impede the generalizability of existing theory. For example, an alternate explanation for one finding, that most effort is devoted to managing bad behavior, may be less due to general features of institutions and more due to Minecraft's demographics, which recall William Golding's *Lord of the Flies*. And generally, as high-stakes as it is for a game server to fail, games are almost by definition lower-stakes than most other applications of institutional theory. Logging out of a server or losing a virtual good are generally less costly than emigrating from a nation or losing a livelihood. But as long as the stakes are high enough that resource scarcity poses a threat to users, tests of general theory in this domain remain justified.

Administrators' motivations to voluntarily incur the many costs of server management are affected by many unobservables that we do not investigate. Similarly, users are driven by many motives in how they choose which servers to explore and commit to, and may not experience the choice, for example, to leave an underperforming server as low or no cost. Nevertheless, we defend a rational choice framework as a model of administrators, the agents analyzed in this work, because of the costs in time and effort they incur by choosing, for whatever reason, to opt into the difficult task of server management.

Our causal interpretation of our finding depends on the assumption that servers cannot maintain a core group if they are struggling with resource availability problems. And such problems are common: a small number of malicious or even naïve users can cripple server performance and drive core users away. Of course, in the absence of random assignment it is impossible to defend a causal argument definitively; it may be that having a large core group causes integrated governance effort, or that third variables like charismatic leadership cause both large core groups and integrated governance. These results are of no less interest if the causal direction we impose is invalid: alternative interpretations are also of immense theoretic interest for the emergent, scalable collective action they imply.

## Conclusion

Online amateur institutions such as game servers, blockchains, wikis, forums, and social networks have become an exciting proving ground for researchers to scale up traditional comparative institutional analyses [43]. But, much more importantly, they are a proving ground for amateurs to develop their leadership abilities, and aptitude for the basic skills of democratic participation. This work illustrates the opportunities that general resource management frameworks and online sociotechnical systems promise each other: the former for foregrounding resource management as a constraint that can unify governance perspectives on online institutions, the latter as a source of population-of-population datasets that improve the quality, quantity, and pace of insights into the nature of effective institutions [35]. At a time when technology is empowering and connecting more people, institutional perspectives on online communities contribute to a unified view of human institutional development—trade treaties to town halls, businesses to bulletin boards, *al hima* to *harambee—*and help more people to benefit from the sciences of social design.

## Supporting information

S1 Text Supplementary information. Containing additional information on the context, constructs, processing, analysis, availability of the data, and references.
(PDF)

## References

[1] Poteete AR, Ostrom E. Fifteen Years of Empirical Research on Collective Action in Natural Resource Management: Struggling to Build Large-N Databases Based on Qualitative Research. World Development. 2008;36: 176–195. 10.1016/j.worlddev.2007.02.012

[2] Ostrom E. A diagnostic approach for going beyond panaceas Proceedings of the National Academy of Sciences. 2007;104: 15181. 10.1073/pnas.0702288104 PMC2000497 17881578

[3] Pateman C. Participation and democratic theory Cambridge University Press; 1970.

[4] Nozick R. Anarchy, State and Utopia. Basic Books; 1974.

[5] Olson M. The Rise and Decline of Nations. New Haven: Yale University Press; 1984.

[6] Scott JC. The Art of Not Being Governed. Yale University Press; 2009.

[7] Ostrom E. Understanding institutional diversity. Princeton University Press; 2005.

[8] Ostrom E. Beyond markets and states: polycentric governance of complex economic systems. American Economic Review. JSTOR; 2010;100: 641–672.

[9] Ashby WR. An introduction to cybernetics. L Chapman Hall LTD 1956.

[10] Ostrom E. Designing complexity to govern complexity In: Hanna S, Munasinghe M, Mohan, editors. Property rights and the environment: Social and ecological issues. Washington, D.C.: The World Bank; 1995 pp. 33–45.

[11] Dietz T, Ostrom E, Stern PC. The Struggle to Govern the Commons. Science. 2003;302: 1907–1912. 10.1126/science.1091015 14671286

[12] Cole DH, Ostrom E. The Variety of Property Systems and Rights in Natural Resources In: Cole DH, McGinnis MD, editors. Elinor Ostrom and the Bloomington School of Political Economy. Lexington Books; 2010 pp. 123–150. 10.2139/ssrn.1656418

[13] North DC. Institutions institutional change and economic performance. Cambridge University Press; 1990.

[14] Powell B, Stringham EP. Public choice and the economic analysis of anarchy: a survey. Public Choice. 2009;140: 503–538. 10.1007/s11127-009-9407-1

[15] Olson M. Dictatorship, Democracy, and Development. American Political Science Review. 2013;87: 567–576. 10.2307/2938736

[16] Boyd R, Borgerhoff-Mulder M, Durham WH, Richerson PJ. Are cultural phylogenies possible? *Human by nature*: *Between biology and the social sciences*. CRC Press; 1997 pp. 355–384.

[17] Scott JC. State Space: Zones of governance and appropriation The Art of Not Being Governed. Yale University Press; 2009 pp. 1–16.

[18] Ostrom E. Design principles of robust property-rights institutions: What have we learned? In: Ingram KG, Hong YH, editors. *Property Rights and Land Policies*. Cambridge, MA: Lincoln Institute of Land Policy; 2009.

[19] March JG, Schulz M, Zhou X. The Dynamics of Rules: Change in Written Organizational Codes. Stanford University Press; 2000.

[20] Akçay E, Roughgarden J, Fearon JD, Ferejohn J, Weingast BR. Biological institutions: The political science of animal cooperation working paper. 2014;: 1–43.

[21] Ashby W. Requisite variety and its implications for the control of complex systems. Cybernetica. 1958;1: 1–17.

[22] Pattee HH. The Physical Basis and Origin of Hierarchical Control *Hierarchy Theory*: *The Challenge of Complex Systems*. George Braziller; 1973 pp. 73–108.

[23] Simon HA. Understanding the Natural and the Artificial Worlds The Sciences of the Artificial. MIT Press; 1996 pp. 1–24.

[24] DeDeo S. Major Transitions in Political Order. In Walker S. I., Davies P. C. W., & Ellis G. F. R. (Eds.), From Matter to Life: Information and Causality. Cambridge University Press; 2017 pp. 393–428.

[25] Heaberlin B, DeDeo S. The Evolution of Wikipedia’s Norm Network. Future Internet 2016, Vol 8, Page 14. Multidisciplinary Digital Publishing Institute; 2016;8: 14 10.3390/fi8020014

[26] Centola D, Baronchelli A. The spontaneous emergence of conventions: An experimental study of cultural evolution. PNAS. 2015;112. 10.1073/pnas.1418838112 PMC4343158 25646462

[27] Gürerk Ö, Irlenbusch B, Rockenbach B. The Competitive Advantage of Sanctioning Institutions. Science. 2006;312: 108–111. 10.1126/science.1123633 16601192

[28] Lansing J, Kremer J. Emergent properties of Balinese water temple networks: Coadaptation on a rugged fitness Landscape. American Anthropologist. 1993;95: 97–114.

[29] Lewis K, Gonzalez M, Kaufman J. Social selection and peer influence in an online social network. PNAS. 2012;109: 68–72. 10.1073/pnas.1109739109 22184242 PMC3252911

[30] Kossinets G, Watts D. Empirical analysis of an evolving social network. Science. 2006;311 10.1126/science.1116869 16400149

[31] Bettencourt L, West G. A unified theory of urban living. Nature. 2010;467: 912–913. 10.1038/467912a 20962823

[32] Ostrom E. Governing the commons: The evolution of institutions for collective action Cambridge University Press 1991.

[33] Guimera R, Uzzi B, Spiro J, Amaral L. Team assembly mechanisms determine collaboration network structure and team performance Science. 2005;308: 697 10.1126/science.1106340 15860629 PMC2128751

[34] Castronova E. On the Research Value of Large Games: Natural Experiments in Norrath and Camelot. Games and Culture. 2006;1: 163–186. 10.1177/1555412006286686

[35] Hill BM, Shaw A. Studying Populations of Online Organizations In: González-Bailón S, Welles BF, editors. *Oxford Handbook of Networked Communication*. Oxford, UK: Oxford University Press; 2017.

[36] Kittur A, Kraut RE. Beyond Wikipedia. New York, New York, USA: ACM Press; 2010 p. 215 10.1145/1718918.1718959

[37] Shaw A, Hill BM. Laboratories of Oligarchy? How the Iron Law Extends to Peer Production. Journal of Communication Wiley Online Library; 2014;64: 215–238. 10.2307/3227338

[38] TeBlunthuis N, Shaw A, Hill BM. Revisiting “The Rise and Decline” in a Population of Peer Production Projects. New York, New York, USA: ACM Press; 2018 pp. 1–7. 10.1145/1979742.1979491

[39] Sadia A, Garg V, McCoy D, Greenstadt R. Honor among thieves: A common's analysis of cybercrime economies. IEEE eCRS 2013.

[40] Kollock P, Smith M. Managing the virtual commons In: Herring S, editor. Computer-mediated communication: Linguistic, social, and cross-cultural perspectives. John Benjamins Publishing Company; 1996 pp. 109–128. Available: 10.1075/pbns.39

[41] Schweik CM, English RC. Internet Success: A Study of Open-Source Software Commons. MIT Press; 2012.

[42] Pitt J, Busquets D, Macbeth S. Distributive Justice for Self-Organised Common-Pool Resource Management ACM Trans Auton Adapt Syst. 2014;9: 1–39. 10.1140/epjst/e2012-01690-3

[43] Krafft P, Keegan BC, Frey S. Designing digital institutions for participatory change. arXiv. 2019. Available: https://arxiv.org/abs/1902.08728

[44] Hardin G. The Tragedy of the Commons. Science. 1968;162: 1243–1248. 10.1126/science.162.3859.1243 5699198

[45] Schmitt P, Swope K, Walker J. Collective action with incomplete commitment: Experimental evidence. Southern Economic Journal. 2000;66: 829–854.

[46] Lazer D, Pentland A, LA A, Aral S, Barabási A-L, Brewer D, et al Life in the network: the coming age of computational social science. Science. 2009;323: 721–723. 10.1126/science.1167742 19197046 PMC2745217

[47] Bainbridge WS. The Scientific Research Potential of Virtual Worlds. Science. 2007;317: 472–476. 10.1126/science.1146930 17656715

[48] Olson M Jr. The Logic of Collective Action: Public Goods and the Theory of Groups Harvard University Press; 1965.

[49] Krebs B. Who is Anna-Senpai, the Mirai Worm Author? In: Krebs on Security [Internet]. 17 Jan 2017 [cited 1 Feb 2019]. Available: https://krebsonsecurity.com/2017/01/who-is-anna-senpai-the-mirai-worm-author/

[50] Lessig L. Code and other laws of cyberspace Basic Books; 1999 Available: http://codev2.cc/

[51] Janssen MA, Holahan R, Lee A, Ostrom E. Lab Experiments for the Study of Social-Ecological Systems. Science. 2010;328: 613–617. 10.1126/science.1183532 20431012

[52] Mason W, Jones A, Goldstone RL. Propagation of innovations in networked groups. Journal of Experimental Psychology. 2008;137: 422–433. 10.1037/a0012798 18729708

[53] Kimbrough EO, Smith VL, Wilson BJ. Exchange, theft, and the social formation of property. Journal Of Economic Behavior & Organization. 2010;74: 206–229. 10.1016/j.jebo.2010.03.017

[54] Putterman L, Tyran J-R, Kamei K. Public goods and voting on formal sanction schemes. Journal of Public Economics. 2011;95: 1213–1222. 10.1016/j.jpubeco.2011.05.001

[55] Becker CD, Ostrom E. Human Ecology and Resource Sustainability: The Importance of Institutional Diversity. Annual review of ecology and systematics. Annual Reviews 4139 El Camino Way, P.O. Box 10139, Palo Alto, CA 94303–0139, USA; 1995;26: 113–133. 10.1146/annurev.es.26.110195.000553

[56] Hannan MT, Freeman J. Organizational ecology Harvard University Press; 1989.

[57] Müller Kapadia, Frey Klingler, Mann Solenthaler, et al Statistical Analysis of Player Behavior in Minecraft. 2015.

[58] Wendel V, Gutjahr M, Battenberg P, Ness R, Fahnenschreiber S, Gobel S, et al Designing a Collaborative Serious Game for Team Building Using Minecraft. 2013 pp. 569–578.

[59] Bukvic II, Cahoon C, Wyatt A, Cowden T, Dredger K. OPERAcraft: Blurring the Lines between Real and Virtual. Georgaki A, Kouroupetroglou G, editors. Proceedings ICMC. 2014.

[60] Leavitt A. Crafting Minecraft: Negotiating Creative Produsage-Driven Participation in an Evolving Cultural Artifact. Selected Papers of Internet Research. Selected Papers of Internet Research; 2013;3: 1–32.

[61] Müller S, Kapadia M, Frey S, Klinger S, Mann RP, Solenthaler B, et al HeapCraft social tools: Understanding and improving player collaboration in Minecraft. 2015.

[62] Fehr E, Glätzle-Rützler D, Sutter M. The development of egalitarianism, altruism, spite and parochialism in childhood and adolescence. European Economic Review. 2013;64: 369–383. 10.1016/j.euroecorev.2013.09.006

[63] Almas I, Cappelen AW, Sorensen EO, Tungodden B. Fairness and the Development of Inequality Acceptance. Science. 2010;328: 1176–1178. 10.1126/science.1187300 20508132

[64] Isaac R, Walker J. Group size effects in public goods provision: The voluntary contributions mechanism. The Quarterly Journal of Economics. 1988;103: 179–199.

[65] Hirschman AO. Exit, Voice, and Loyalty: Responses to Decline in Firms, Organizations, and States. Harvard University Press; 1970.

[66] Dasgupta A, Beard VA. Community Driven Development, Collective Action and Elite Capture in Indonesia. Development & Change. Blackwell Publishing Ltd; 2007;38: 229–249. 10.1093/wbro/15.2.225

[67] Warren C, Visser L. The Local Turn: an Introductory Essay Revisiting Leadership, Elite Capture and Good Governance in Indonesian Conservation and Development Programs. Hum Ecol. Springer US; 2016;44: 277–286. 10.1007/s10745-016-9831-z 27445425 PMC4937090

[68] Hirschman AO. Exit, Voice, and the State. World Politics. 1978;31: 90–107. 10.2307/2009968

[69] Hess C, Ostrom E. Understanding Knowledge as a Commons: From Theory to Practice. Cambridge, MA: MIT Press; 2007.

