# Supplementary material for: Emergence of integrated institutions in a large population of self-governing communities
Source: PLoS One. 2019 Jul 11;14(7):e0216335. doi: 10.1371/journal.pone.0216335 (PMC6622466; doi:10.1371/journal.pone.0216335)
Supplement: S1 Text — Containing additional information on the context, constructs, processing, analysis, availability of the data, and references. (PDF) [file pone.0216335.s001.pdf]

Supplement for

Emergence of integrated institutions in a large population of self-governing communities

Seth Frey*,[1,2], Robert W Sumner[3]

[1] Communication Department, University of California Davis, Davis, CA, USA

[2] Neukom Institute for Computational Science, Dartmouth College, Hanover, NH, USA

[3] Computer Science, ETH Zurich, Zürich, Switzerland

Contents

— Supplementary text
  — Data context
  — Data constructs
  — Data processing
  — Data analysis
  — Data availability
  — Supporting references

Supplementary text

Data context

Self-hosting on the Internet

A server is a computer that provides services that other computers can access through the physical network structure of the Internet. These services can be of many types, and while the most familiar type of service is of web pages to the World Wide Web, other services support other modes of interaction. For example, multi-player game servers can foster social interactions in virtual worlds. Even though most of our interactions on the Internet are through professionally managed servers, the barriers to amateur servers are low, and it is increasingly common for public online community services to be hosted by non-professionals with only a minimum of skill at server management. Amateur hosting brings the time and money costs of maintenance to a server's main administrator, likely an individual with access to limited resources. Any efforts that that administrator makes to solicit resource provisioning assistance from users makes their behavior relevant to questions of governance posed by the frameworks of self-organizing resource management.

Minecraft

Minecraft is a multi-player virtual world game well-known for its open unplotted structure and immense popularity: it is the second best selling of all time behind Tetris. It stands out from other games as a domain for institutional research, not for anything about the internal features of the game as a game, but for the culture around it and technical features of how it is made available to its players. Unlike most other multiplayer games, which are hosted on central severs controlled by the game developer, Minecraft permits a decentralized hosting model in which amateurs host instantiations of the game on servers they manage.

This study is based on data from a continuous survey of data from 300,000 Minecraft server IPs over two years. Regarding privacy, we only accessed data that servers made public. By default, servers provide basic information about their current state through an API that is publicly accessible via the Internet. Also by default, they have no terms of use governing their APIs, although some deviations from this default are likely, for those servers that may have published terms of their own.

It was not feasible to determine which might have published terms, much less whether any terms had provisions that were relevant to how we scraped. However, we only accessed that data that was provided publicly by each server's API, subject to whatever constraints that server might have imposed on our queries; if a server did not provide a datum through its API, we did not record it.

Minecraft players

From what studies exist, scientific and otherwise, we can build a coarse sense of the demographics of the Minecraft user base. We estimate that approximately 80–90% of players are male and that the median player is a young adult (age 19) [70,71].[1] Out of the global community of users, the majority are based in the US and northern Europe. We do not have demographic statistics on the subset of players who are also administrators, but because of the technical skills and monetary costs required to administer a server, administrators are likely slightly older than other players.

Another dimension of social context to consider is that users are often acquainted in real life. One study of smaller game teams found that 75% of virtual colleagues are real-world friends or family [72]. By reducing anonymity and freedom from consequences for antisocial behavior, real world acquaintance raises the accountability of users to each other, and may to some extent supplant the function of installed rules. We predict that that non-observable informal avenue for norm enforcement is more likely in smaller communities, which provides an alternate, but non-competing hypothesis for our finding that software rules increase in number with population maximum.

Minecraft servers

To host a Minecraft community, a prospective administrator rents or buys access to a server, installs and configures the game's server software, installs plugins, and may then log in. When the server is started, a unique world is generated from the "seed" of a single number, usually randomly generated.

Administrators create rules and other modifications to their server by installing plugins. Plugins are small programs that are designed to modify server functionality in a well-defined way. Administrators may certainly deliberate with their users before installing new plugins, but they are

[1] for the results of an informal community solicited survey conducted in 2011, see http://i.imgur.com/xmL5Y.png

ultimately free to act unilaterally, and typically do. While many plugins add frivolous functionality (in the literal sense that they foster play), many are explicitly developed to aid in the governance of specific resources, either by implementing single rules (like narrow proscriptions on possible behaviors) or larger self-contained institutions (such as markets or social hierarchies). While play is an interesting subject, the focus on this work is on the resource-related social dilemmas that administrators overcome in the process of supporting play and other user interactions.

Administrators must then attract a user base. They may recruit from among personally known peers, and they often advertise their community at a larger scale on web sites that provide users with searchable lists of servers, explained below.

Minecraft ecosystem

Server lists and the administrator community. Communities compete for users through "server list" sites, which players use to search for communities that match their interests. These sites work by aggregating URLs and other data from hundreds of thousands of active servers.[2] To improve the richness of this system, developers devised a public query API that game servers use to expose basic realtime statistics about the state and health of their community. This information includes a server's version number, its currently active population (with unique public user IDs), its maximum population, and so on. Server list sites are constantly querying game servers for this information in order to provide quality signals about each community they list. This query API information is made available by default on all servers, and it is the basis of our information about the governance style and success of each server in the corpus.

Because servers compete for users, and because users can join and leave voluntarily at low or no cost, the Minecraft ecosystem unwittingly implements the "market for tyrants" theory of utopia described by Robert Nozick in *Anarchy, The State, and Utopia* [4]. We use this characteristic to explain why observed governance characteristics in our population may reflect the institutional

[2] such as http://minecraftservers.org

preferences of users rather than those of administrators. Others have shown that even leaders with a history of corruption can be incentivized to work in the interests of those they serve [73].

Plugin sites and the developer community. Use of server lists, and competition between servers, are dimensions of the larger ecosystem of code, culture, and communities within which each server operates. Another dimension important to this study are "mod" sites[3] where developers have converged to create a central repository of open source plugins.

In games such as Minecraft, collections of plugins grow into fully fledged ecosystems as a platform's power users coordinate in the programming and free distribution of useful tools. The broader Minecraft community had developed almost 20,000 plugins by 2016, although the top 500 accounted for nearly 90% of plugins in use.

To help administrators make sense of the large number of modifications, developers are required to assign each plugin they write to at least one pre-specified category. We use these categories as the basis of rules types, described in full detail in the Data Constructs section.

Data constructs

Administrators

One fundamental assumption of our analysis is that administrators are motivated to build community and overcome the social dilemmas that attend community building and resource sharing. We assume that they pursue this goal by means they consider effective. Server administration is costly in time and money, and the game can be played in a "single-player" mode without a server, so we infer that administrators go to the additional difficulty of enabling social play over the Internet because it is worth the costs.

By contrast to administrators, normal players' motivations are more opaque and varied. For this reason, we structured the analysis so that our conclusions require boundedly rational choice from administrators, not users.

[3] such as https://mods.curse.com/bukkit-plugins/minecraft/

A less vital assumption is that each server has a single administrator, and that that administrator monopolizes administrative decision-making. While this structure is customary, it is not mandatory. It may be that administrators provide administrative access to multiple players, or that they consult their users before instituting changes. We cannot observe this, but because a server can ultimately only be run one way, the outcome chosen by a group of decision makers is still a comparable to that chosen by a single decision maker, and it is safe to model that group as if it is a unitary agent.

Size

There are many ways to define the size of a community, such as its number of monthly visits, number of unique visits, or return visits. Instead, we use the population maximum, the value of a server parameter that limits the maximum number of simultaneous users. A downside of the population maximum parameter is that it underestimates other more intuitive definitions of size. For example, a server with a maximum of 10 simultaneous may still be visited by thousands of unique players in a month. However, this measure also has advantages:

— Unlike other potential measures, which are based partly on the results of an administrator's behavior over time, population maximum is set directly by the administrator upon installing the server. It captures an administrator's intentions for their server.

— Servers can handle indefinite load when that load is distributed sparsely over time. A server starts to encounter its performance limits only as it struggles with simultaneous requests. These performance limits are due to finite CPU, RAM, and bandwidth (some of the resources that administrators manage). The population maximum is a satisfactory definition of server size in part because it is directly subject to resource constraints and must be set with them in mind. Administrators must balance their desired community size against their server's performance limits, and an administrator's choice to aspire toward a large community comes with the knowledge that they must arrange for sufficient resources to give that number of simultaneous users a good experience. The consequences of under-provisioning a high traffic server are network lag, low frame rate, unsynchronized interactions, lost connections, and other features that are known to quickly turn users away. Lag in particular, often referred to as "lagg" within video game communities, is a notorious turn-off, and is a major target of malicious agents seeking to disrupt a server.

— Because it must be manually updated, a server's population maximum is likely to remain stable and unchanged over months. When this important parameter does change, it is because the administrator intentionally changed it.

— Although it is very different in definition from the core group variable, population maximum also puts a soft upper bound on core group size. Out of 5,216 servers, we observe only 2 instances in which a community's core is larger than its maximum number of simultaneous users.

— Administrators do have incentives to set it honestly. If they set it lower than they desire, it will not be possible to host as many users as they desire. If they set it higher than they desire, they risk a sudden influx of users crashing their server or degrading the quality of game service below acceptable levels.

Community success

We evaluate servers in terms of the success of their administrators in recruiting a core group of committed community members. We define success specifically as the number of users who returned to that community at least once a week for a month. In contrast to raw number of visits over a time period, intermittent returns indicate a sustained level of interest and commitment in an environment in which many other communities are competing for each user's attention. Months are meaningful time units because they define the customary billing cycle for server hosting, because it takes about a month to bring a community to a mature state of development by the game's constructs, and because most servers endure for only 0–3 months.

With this measure of success, the basic unit of analysis is the server-month. An alternate quantification of community might have focused on the number of people who visited or returned over the entire lifetime of that community. However, because communities varied widely in their lifetimes, focusing on server-months improved the comparability of the longest to the shortest lived communities.

To make success relative to administrators' different goals, our figures consider each server's core group size plotted against its maximum population, and our models capture other interactions between these variables.

Unit of analysis

The servers in our sample varied widely in the number of weeks that they persisted. In order to control for the relative over-representation of longer lived servers (because they endured for more server months), we aggregated the data down to the scale of server-months and completed our dataset by selecting the most successful month of core group activity in the lifetime of each server. By selecting the month that a server reached its peak core group we improved the comparability of administrators by comparing them at their peak community building performance. This is the month

in each server's lifetime when it is safest to assume that the administrator is highly motivated to accomplish the goal of building community. The cost of this choice is that this work fails to take advantage of the longitudinal nature of our raw dataset, a task we leave to future work. Alternative approaches would have been to select a random month, the very first month, or the very last month, or to control another way for the differences in sampling density of each server/administrator (and correlation of sampling density with success via longevity).

Resource types

Understanding a community's resources in these abstract terms makes community success amenable to frameworks for analyzing real world community resource management institutions such as local fisheries, irrigation systems, and forest management communities. In the case of Minecraft communities, administrators must work to manage public goods and common pool resources of three types: physical, virtual, and, more abstractly, antisocial behavior, a type of pollution.

Physical resources. The administrator usually has exclusive or de facto exclusive access to their server's overall status, underlying settings, and total available resources. Resource intensive applications such as Minecraft require increasing amounts of CPU, RAM, disk, and network bandwidth for each additional user on the server. Because an administrator, by default, cannot keep a player from logging on, a server's computing resources can be classed with common pool resources or congestable public goods. And because Minecraft defines a large, dynamic, real-time, 3D virtual world, these resources are very easily stressed, with standard recommendations prescribing about 5 GB of disk space, 1–3 Mbits/s of up- and download bandwidth, and 1 GB of RAM *per player* in order to provide players an acceptable level of moment-to-moment responsiveness.[4] These sometime prohibitive requirements can themselves quickly become inadequate in the presence of malicious or even naïve users who intentionally undermine service by triggering resource-intensive game events.

[4] see, for example, https://minecraft.gamepedia.com/Server/Requirements/Dedicated

When computational resources are inadequate, the server will become unresponsive to player actions. This is called lag, and players are sensitive enough to it that it need only persist for a few minutes for them to leave for another server.

Additionally, administrators require monthly fees to maintain their connection to the Internet, whether hosted privately or through a firm. While it is possible to pay nothing to provide a public server, the typical amateur will begin paying about $10USD/month, and an administrator of a large, popular community may pay hundreds per month. While these figures may not sound like much compared to the millions invested in fisheries, for example, it is worth remembering that administrators are usually volunteers, and often teen-aged, and this study focuses on non-professional servers. Administrators are responsible for the payment of these fees by default, at which point the server itself is being provided as a public good to its players. When administrators attempt to raise fees from players, they are engaging with the well known problem of provisioning a public good. Players are incentivized to free-ride (it is, indeed, the norm), but failing to provide computing resources at sufficient quality or capacity undermines players' experiences and makes them likely to leave a community.

Virtual resources. Minecraft itself defines many virtual resources. We focus on in-game common pool resources that require both provisioning effort and responsible extraction. These are chests, crops, trees, and mobs. All of these are by default non-excludable, subtractable, and take effort to provide. Many plugins assist in the management of these resources, some by creating the idea of private property rights, others by improving logging of game events.

Bad behavior. Bad behavior is common enough in Minecraft that it is referred to within the community as "grief." Most (but not all) servers admonish players to not grief each other. Because the game's world is so open-ended, users can find many creative and hard-to-detect ways to harass each other. Harassment can take the form of chat-based insults or virtual violence, hacking a server, cheating by covertly installing performance aids on the client side, vandalism of the work of others, cursing, or otherwise behaving outside the bounds set by the administrator. Bad behavior can also

affect server performance: malicious players will commonly start large virtual fires, grow a large virtual animal populations. These dynamic game entities require computational resources to represent individually, so if an attacker can arrange them to grow exponentially, even for a brief period, they can effectively deny service to the rest of the community.

The plugins of the Minecraft ecosystem provide a very wide range of partial solutions to the problems of bad behavior. These include temporary bans, full exile and blacklisting, hacking counter measures, cheater detection, peer monitoring and reporting tools, admin surveillance tools, distribution of authority to trusted members, and tools for keeping and restoring backups after attacks.

No resource. Many plugins in use by servers did not identify a resource, or did not identify themselves as relevant to governance. We excluded these from the analysis, except in the form of a control variable that tracked the raw number of plugins separately from the number of resource related plugins.

Rule types

We describe four types of software rule system recognized by the plugin developer community for addressing these various resource problems: "chat," "informational," "economy," and "admin tools." These strategies are not only common in Minecraft, but are recognized in the study of resource management generally.

Chat plugins are those that facilitate inter-player communication, either by expanding built-in capabilities, creating new communication channels, or improving the interoperability of multiple communication schemes. By default, users can only communicate via text chat, but various plugins enable the higher bandwidth of voice chat. Rules that improve communication have a recognized role in frameworks for institutional analysis [8], and unconstrained communication is known to be valuable in many resource governance settings [51], including Minecraft, which has plugins to support VOIP platforms such as Skype, Discord, and Teamspeak. In one case we observed, a server with a 16+ age limit verified compliance with that limit by requiring all users to communicate via voice chat.

Informational plugins are those that aggregate data for users and administrators and improve their knowledge of strategic decisions. Information rules are another type of rule that are recognized as important in frameworks for institutional analysis [8]. In Minecraft, the informational plugin Dynmap[5] help users monitor each other's locations and activities.

Economy plugins install private property rights and exchange mechanisms to permit players to trade in both virtual and physical resources. These plugins function as self-contained institution modules that administrators can use to ensure that certain resource distribution problems solve themselves. While most economy plugins restrict themselves to the distribution of virtual resources, some economy plugins, like Buycraft[6], help administrators cover server fees by selling social status or game objects.

Administrative tools are plugins that increase beyond the default the actions that the administrator can perform. These plugins help administrators exercise greater control over server state and player behavior. For example, the popular OpenInv[7] plugin allows administrators to covertly search and remove (potentially forbidden) belongings from their users' inventories.

It is important to note that not all governance need happen through plugins. If the members of a server's core group maintain real world relationships, they may have access to the much more compelling range of enforcement tools made available by physical co-presence. This is especially likely in the smallest server communities (of four and fewer), which are more likely to be managed by pre-existing real-world peers who could in principle resort to physical enforcement of their server's virtual rules. This caveat imposes some nuance on how we compare success in large and small communities, but to the extent that non-virtual enforcement characterizes small-community success, our key results are more rather than less likely to hold. In particular, this would not change our results for large servers, nor our observation that success looks different in small versus large servers.

---

[5] http://mods.curse.com/bukkit-plugins/minecraft/dynmap
[6] http://mods.curse.com/bukkit-plugins/minecraft/buycraft
[7] http://mods.curse.com/bukkit-plugins/minecraft/openinv

Data processing

We compiled a list of 376,576 Minecraft server addresses by querying for IPs from multiple sources[8] over two years. After filtering for communities that were minimally viable and minimally comparable, our final dataset was 5,216. We also identify, within these, the 1,837 minimally successful communities.

Minimally viable

Of 376,000 servers, only 157,747 ever responded to our queries, and only 91,271 remained live for at least the span of one minimally costly month.

Minimally comparable

Our comparative analysis of governance schemes across communities required them to share a minimum number of common characteristics. All communities had to have a valid API, keep their functioning close to default, and report full community and governance data. This step reduced our sample from 91,271 to 5,216 server communities.

API is trustworthy. Servers' APIs are used to communicate information that prospective users use when deciding whether to join. But servers are customizable enough that administrators can undermine the ability of the platform's API to correctly report its performance statistics. We filtered out servers that were using special plugins that falsify this data in order to present more appealing statistics to players and server lists. We also filtered out servers reporting impossible values, such as a maximum population of zero or less.

"Vanilla". The world of Minecraft, being virtual, is virtually unlimited in its customizability. Indeed, with very simple modifications, a developer can rewrite physics and define away the finiteness of most of the finite resources at the core of this study. Finite or non-excludable resources can be made infinite or excludable, and therefore no longer susceptible to the collective action problems we study.

---

[8] http://minecraftservers.org
https://www.reddit.com/r/mcservers/ and
https://www.shodan.io/search?query=+port%3A25565

Deviations from vanilla play undermine key premises of this work, not to mention the comparability of servers to each other, and the reliability of their API statistics. Fortunately, and surprisingly, many administrators, especially those running small-scale amateur servers, choose to keep the game close to its default state, with resources that are indeed limited and in need of active management. They do so despite the "god-like" power that they have to define resource problems away entirely. Within the game community, servers whose mechanics remain close to default are called "vanilla" or "semi-vanilla" servers.

It is relatively straightforward to determine how far a server has drifted from vanilla play, as the most important deviations are implemented by easily identified plugins that we incorporate into our exclusion criteria. For example, some administrators install plugins that link many worlds into larger collections of servers. In these "multiverse" servers, API statistics fail to capture the extent of an administrator's community, so we also excluded them from our analyses.

Full reporting. The basic API reports statistics such as a server's maximum simultaneous users and list of users present. Beyond these basic statistics, administrators can opt in to a more full version of the API in order to more effectively compete for users. It is this non-default version of the API that reports a server's installed plugins, which we required in order to characterize a server's governance regime. Only about 10% of servers use the full version of the API. Because enabling the extended API improves the transparency of a community to prospective users, this filtering step may amplify the bias in our analysis for administrators who are motivated to recruit widely for visitors and core members. This source of bias would seems more likely to support than undermine our conclusions, particularly because it provides an additional filter for administrators that are highly motivated to overcome resource challenges toward building a devoted community.

Minimally successful

An additional criterion that we included for a subset of our analyses is that a server was "minimally successful," with a core group of size greater than one. We used this subset of 1,837 out of 5,216 servers to test governance factors against group size. We did this because we did not want unsuccessful servers to drive the results of our tests, and we wanted to be able to interpret results over

size as valid across the range a minimally successful servers. We did not include success as a covariate because our tests of success were already using population maximum as a covariate, and because administrators have more control over the value of the population maximum parameter than over then number of users that become community members. To manage the issues surrounding multiple tests of interacting constructs, we followed the procedure for multiple testing laid out by mediation analysis. Starting with models of population maximum, we moved to models of core group size that included population maximum as a control.

Data analysis

Our central statistical tests regress core group size against four measures of regime complexity and the interactions of each with server target size, and several basic covariates. After describing the variables we describe the tests.

Variables

DVs

— *Core group size.* This is the number of people who visited the server at least once a week during a server month. This is a main dependent of interest.

— *Population maximum.* This is the maximum number of users allowed simultaneously on a server. It is set by the administrator. Because some users set this astronomically high (enough to throw off our analysis), presumably to signal that they did not intend an upper limit, we capped this value at that maximum number of users ever observed simultaneously on any server. We $\log_2$ transform the variable in our models (precisely $log_2(x+1)$, to theoretically allow for maximum population limits of size zero).

IVs: Controls

— *Week.* To capture large-scale fluctuations in the popularity of Minecraft, we included as a covariate the number of weeks from the Unix epoch, scaled.

— *Weeks up.* To capture potential effects of a server's age on its governance characteristics, we included a term for the number of weeks between the server month of interest and the date that our scraper first contacted the server.

— *API richness.* Servers vary in how much information they make available through various APIs, beyond the minimum required for our study. We included it in our models because it may be related to the willingness of users to visit a server, or to the level of involvement of the administrator.

— *Software count.* This is the number of plugins installed on the server, whether or not they are related to governance. This is in contrast to the rule count variable below, which counts only governance related plugins.

— *Population maximum.* Described above as a dependent. In addition to being a dependent of interest in itself, we also use this variable as a covariate and interaction variable in models of the other dependent, core group size. Because it effectively puts an upper bound on the size of a community's core group, the two are likely to be correlated.

IVs: Governance variables

— *Rule count.* This is the number of rules that are involved in the governance of some type of resource. It equals the sum of rules by resource type. It does not equal the sum of rules by rule type, because some rules are categorized to have multiple types, and some have none. Of course, some plugins install many rules, and some are very simple, but rule count nevertheless provides a proxy for the size of a server's governance infrastructure.

— *Rule diversity.* We identify plugins in terms of four rule types. Some servers use many rules of many types, while others use a smaller variety of types. We capture diversity of types in a variable by counting the number of types of rules represented on a server. This number of types is known in ecology as variety. We use variety instead of entropy because many servers have a very small number of rules, and entropy is known to be sensitive to small sample sizes and bins of size zero.

— *Rule scope (or "resource diversity").* Rule scope is the range of resource problems that a server is using plugins to address. We use the ecological variety, number of types, for the same reason that we use it to quantify rule diversity.

— *Rule specialization.* Because plugins are made publicly available, many communities use many of the same plugins, with the most popular plugins in use on tens of thousands of servers. The rule specialization of a server was the median of the number of other servers also using each plugin it was using. In our models we actually used the inverse of this count in order to define servers without any plugins as having rule specialization equal to zero. This fourth variable is insignificant in all tests. Exploratory analysis shows that its effects are accounted for by the software count control in single variable tests, and likely also by rule count in the full models.

Model Specifications

We used a mix of univariate and multivariate models on two dependent variables to understand the relationship between size, success, and various governance variables of interest. For all of these the unit of analysis was the server-month, with the dependent variables being the size and success of a server (population maximum and core group size). Though we do not characterize our analysis in terms of the statistical concepts of mediation or moderation, we followed the procedure established by moderated mediation analysis for testing over multiple dependent variables, first testing effects on population maximum, then testing effect on core group size with population maximum as a control variable and in interaction terms. Tables 1, 2, and 3 describe our models and their effects.

We modeled the logarithm of population maximum in five regressions, one that fit Week, Weeks up, API richness, and Software count, as well as Rule count, Rule diversity, Rule scope, and

Rule specialization, and four "single variable" models that fit the controls with just one of each of the governance variables. We used this range of tests to help us understand the relationship of the governance variables with each other. For example, rule scope is significant when modeled alone, but its effects seem to be accounted for by a combination of the other three (Table 1). And the positive coefficients for rule diversity alone become negative when considered in the context of the other variables, suggesting an overall increase in the diversity in rules with size, caused by the other variables, that is strong enough to mask a net decrease in diversity when they are all taken into account (Table 1). All models of population maximum were conducted on the subset of minimally successful communities — those with core groups > 1. This eliminates the inordinate influence of failed communities on our tests, and permits us to interpret any effects of size in terms of successful communities only.

We then modeled core group size with the same terms as above, plus the logarithm-plus-one of population maximum and its interactions with each of the four governance variables. Means before centering of each of the interacted variables were as follows: *Population maximum=3.17, Rule count=2.17, Rule diversity=1.36, Rule scope=0.76, Rule specialization=0.0059*. As above, we fit one full model with all four governance variables and four more models fitting each governance variable alone. Because core group size is a count of events — arrivals on a server — and because the baseline rate of these events varies by server, we used a negative binomial regression, in which parameter theta is understood as a dispersion over the lambdas of a population of Poisson processes.

For both the models of population maximum and core group size, Chi-squared tests of the full models against controls-only models showed a significant difference in variance explained, while tests of the single-variable models against the control models varied in their ability to exceed the $p<0.001$ threshold.

To better understand the rule count, rule diversity, and rule scope effects, we ran four more models (Tables 3–4),

— One linear regression fitting the four types of rule, plus a software count control, against population maximum.

— One negative binomial regression fitting the above variables plus population maximum and its interactions with the four types against core group size.

— One linear regression fitting the three types of resource, plus a software count control, against population maximum.

— One negative binomial regression fitting the above variables plus population maximum and its interactions with the three types against core group size.

Plots

The plots show the same data as fit to the models, except that we do not display "singular" bins containing only one server. This visualization choice led us to exclude two "above diagonal" outlier communities that were occupying two above diagonal bins. Including these does not influence the apparent conclusion of the plots, our analyses, or the manuscript. We excluded them to facilitate quick visual inspection of the complex 2D histogram plots that dominate our figures.

Data availability

The data are available at https://dash.ucdavis.edu/stash/dataset/doi:10.25338/B8Q88S

Supporting references

70. Canossa A, Martinez-Hernandez J, Togelius J. Give Me a Reason to Dig. ACM FDG. 2013. Available: http://dl.acm.org/citation.cfm?id=2282400

71. French DJ, Stone B, Nysetvold TT, Hepworth A, Red WE. Collaborative Design Principles From Minecraft With Applications to Multi-User CAD. ASME; 2014. doi:10.1115/DETC2014-35279

72. Williams D, Ducheneaut N, Xiong L, Yee N, Nickell E. From Tree House to Barracks: The Social Life of Guilds in World of Warcraft. Games and Culture. 2006;1: 338–361. doi:10.1177/1555412006292616

73. Avis E, Ferraz C, Finan F. Do Government Audits Reduce Corruption? Estimating the Impacts of Exposing Corrupt Politicians. Cambridge, MA: National Bureau of Economic Research; 2016 Jul. doi:10.3386/w22443
